# Young migrants’ sexual rights in Sweden: a cross-sectional study

**DOI:** 10.1186/s12889-021-11672-1

**Published:** 2021-09-06

**Authors:** Mazen Baroudi, Anna-Karin Hurtig, Isabel Goicolea, Miguel San Sebastian, Robert Jonzon, Faustine Kyungu Nkulu-Kalengayi

**Affiliations:** 1 Department of Epidemiology and Global Health, Umeå University, Försörjningsvägen 7D, SE-907 37 Umeå, Sweden; 2 The Public Health Agency of Sweden, Nobels väg 18, SE-171 82 Solna, Sweden

**Keywords:** Young migrants, Sexual rights, Sexual health services, Non-binary, Sweden

## Abstract

**Background:**

In national public health surveys including those assessing sexual and reproductive health, migrants generally tend to be underrepresented due to cultural, linguistic, structural and legal barriers, minimising the possibility to measure sexual rights’ fulfilment in this group. This study aims to describe to what extent sexual rights of young migrants in Sweden are being fulfilled.

**Methods:**

A self-administered questionnaire was used to collect data from 1773 young (16–29 years) migrants by post, online, and at language schools and other venues. Sexual rights were operationalised and categorised into five domains adapted from the Guttmacher-Lancet Commission’s definition. These domains included the right to: 1) access sexual and reproductive healthcare, 2) access information and education about sexuality and sexual and reproductive health and rights, 3) have bodily integrity, 4) make free informed decisions about sexuality and sexual relations and 5) have a satisfying and safe sexual life. Descriptive analysis was used to assess the extent of fulfilment for each right.

**Results:**

There were wide variations in the fulfilment of sexual rights between subgroups and among the five domains. Most respondents rated their sexual health as good/fair, however, 6.3% rated their sexual health as bad/very bad. While most of those who visited related services were satisfied, 17.4% of respondents refrained from visiting the services despite their needs. Around four in ten respondents did not know where to get information about sexuality and sexual health. One-fourth of respondents reported sexual violence. Another 12.7% were limited by family members or fellow countrymen regarding with whom they can have an intimate relationship. Most respondents were satisfied with their sexual life, except for 11.9%. Men, non-binary respondents, lesbians, gays, bisexuals, asexuals, those who were awaiting a decision regarding residence permit and those born in South Asia reported poor sexual health to a greater extent and fulfilment of their sexual rights to a lesser extent than other groups.

**Conclusions:**

Timely and culturally adapted information about sexual rights, gender equalities, laws and available services in Sweden should be provided in appropriate languages and formats in order to raise awareness about sexual rights and improve access to available services. Tailored attention should be paid to specific vulnerable subgroups.

**Supplementary Information:**

The online version contains supplementary material available at 10.1186/s12889-021-11672-1.

## Background

Young migrants from non-European countries are a rapidly growing group in Sweden. In 2019, around one fifth of all youth in Sweden were foreign-born [1]. Young migrants in this study are defined as young people aged 16–29 years who left their home countries to live/settle in Sweden. By December 2018, the most common countries of birth for non-European young migrants living in Sweden in decreasing order were Syria, Iraq, Afghanistan, Somalia, Eritrea, Thailand and Iran [1]. Regardless of the reason for migration, young migrants are generally vulnerable to all forms of violations of their rights, including sexual rights [2–4]. Furthermore, those who move without parents or guardians and those who depart from society’s norms regarding ethnicity, gender, gender identity or expression, functionality and sexual orientation are at increased risk of violations of their sexual rights [2, 5]. Moreover, young migrants often face racism, xenophobia and discrimination as well as marginalisation due to language barriers, a lack of familiarity with the host country’s culture and a lack of knowledge of their rights in their new country [2, 6]. Their vulnerability to violation of their sexual rights may also be exacerbated by poor knowledge about sexuality and rights and how to access information, support and care in host countries [2, 7, 8]. The violation of sexual rights of young migrants not only violates human rights but can also have devastating consequences for the dignity, psychological and personal well-being, self-esteem, independence, reproduction, sexual relations and health of those affected [2, 3, 9].

### Sexual rights

The definitions of sexual health and rights have evolved and are often combined or overlap with those of reproductive health and rights in the literature to form the components of sexual and reproductive health and rights (SRHR). However, these components are presented as conceptually distinct to the extent possible in the Guttmacher-Lancet Commission [10] and World Health Organization (WHO) [11] reports in order to highlight the need to protect sexual rights, which are also the focus of this paper. In this paper, sexual rights are defined as:

“*the right for all people to make free informed decisions over their body, sexuality and relations and to have a safe and satisfying sexual life free from stigma, discrimination, coercion and violence and the right to information, education and health care that gives the opportunity to reach the highest attainable sexual and reproductive health on equal terms”* [10–12].

In other words, a fundamental prerequisite for achieving the highest possible standard of sexual health is that the sexual rights of all people, including migrants, be respected, protected and fulfilled and that all persons respect the rights of others [11]. The Guttmacher-Lancet Commission and the WHO reports further describe sexual rights as human rights principles applied to sexual health. These human rights are already recognised in international and regional human rights documents and other consensus documents, and in national laws [10, 11]. In Sweden, sexual and reproductive rights are formalised in various laws, regulations and the non-discrimination principle, which guarantee all people, regardless of sex, age, ethnic background, religion, disability, gender identity or sexual orientation, the right to control their own body and sexuality without discrimination, violence or coercion and the right to a health system that provides the same opportunities for all people to achieve the best possible sexual health on equal terms [12].

Studies on young migrants in the field of SRHR are sparse, both nationally and internationally. Existing research shows that they faced increased risk of violence and violation of their human rights, including sexual rights, similar to that faced by adult migrants during the migration process (before, during or after), which negatively affected their sexual and reproductive health (SRH) [2, 13, 14]. Young migrant women are considered to be the subgroup most vulnerable to sexual, gender-based or honour-related violence (HRV) as well as violations of their sexual rights in the form of early and forced marriage, sexual exploitation, family and intimate partner violence, and genital mutilation/cuttings [2, 3, 15]. The recent refugee crisis in Europe has also drawn attention to the issue of the sexual exploitation of unaccompanied asylum seekers and refugee boys or young men who are separated from their parents or families. Young migrant men are often a forgotten group when it comes to the prevention of sexual violence, even though they make up a significant share of unaccompanied children, both globally and in Sweden [16–18]. Young lesbian, gay, bisexual, trans and intersex (LGBTI) refugees and asylum seekers are likely to experience multiple abuses, discrimination and marginalisation as a result of the intersection of age, gender identity, sexual orientation, sex characteristics and migrant status/ethnicity, which put them at an increased risk of physical and mental distress [19] and high-risk sexual behaviours [20]. In addition, the available health services in receiving countries rarely meet their healthcare needs [21, 22]. However, there is a need for more research to better understand the intersections of different dimensions of socio-cultural identities and its impacts on the rights of young LGBTI migrants.

Previous Swedish national studies that have assessed SRHR revealed inequities among the Swedish population with regard to sex, gender expression, sexual orientation, age, functionality, educational level, country of birth (migrant status) and length of stay in Sweden [23–26]. Moreover, young migrants living in Sweden represent a heterogeneous group that includes people from different parts of the world, with different backgrounds (sex, gender identity, sexual orientation), experiences, reasons for coming to Sweden, legal statuses and conditions, all which can result in different opportunities to enjoy sexual health and rights and access available services [4]. On the other hand, migrants generally tend to be underrepresented in national public health surveys including those assessing SRHR due to barriers related to language, culture and immigration status. Against this background, Umeå University was commissioned by the Public Health Agency of Sweden to conduct a survey among young migrants. While this paper focuses on sexual rights among young migrants, the project is part of national efforts to examine the situation of sexual and reproductive health and rights (SRHR) in the whole Swedish population.

### Aim

In this paper, we aimed to describe to what extent the sexual rights of young migrants living in Sweden are being fulfilled using five domains adapted from the Guttmacher-Lancet Commission’s definition of sexual rights.

## Methods

All methods were carried out in accordance with relevant guidelines and regulations. For more details, please see the ethical declarations.

### Study design, setting and population

A cross-sectional survey was conducted using a questionnaire that was developed based on two national surveys: the sexual and reproductive health and rights in the general Swedish population (SRHR2017) and the sexuality and health among youths in Sweden (Ungkab15) [24, 25]. The survey was designed in Swedish and English and then translated into Arabic, Dari, Somali and Tigrinya. A pilot study was conducted with around 30 participants in a Swedish-for-foreigner language school to check for potential misinterpretations due to language or cultural differences. After performing language and cultural adaptation, the surveys were back-translated and cross-checked with the English version to ensure accurate meanings. We targeted young people aged 16 to 29 years living in different parts of Sweden and who were born outside Sweden, the European Union member states, North America and Australia, without consideration of their length of stay, reason for migration or legal status.

### Data collection

**Fig. 1:**
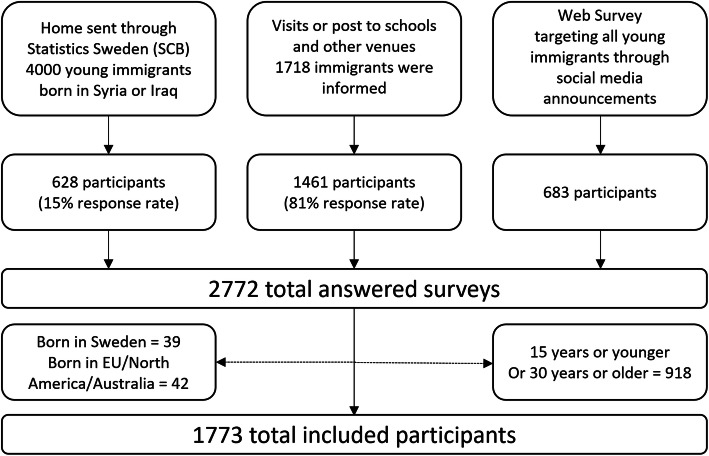
Study participants and data collection methods

The data were collected from March to September 2018 throughout Sweden using three data collection modes: via mail sent to homes by Statistics Sweden (SCB), face to face through visits to schools or post sent to schools and other venues, and via a web survey (Fig. 1). The latter two data collection modes (face to face and web survey) targeted all migrant groups. But due to budget restrictions, the mail survey was only administered to participants from the two most common countries of birth of young migrants namely Syria and Iraq. In total 1773 participants are included in this analysis.

Written informed consents were collected after detailed information about the study was provided to the respondents. It was also stressed that participation was voluntary, respondents could withdraw from the study at any time without consequences, their responses would be treated confidentially and that the results would be reported anonymously.

### Data analysis

#### Background variables

The sample characteristics were described using the following variables: *Ever had sexual intercourse*: yes/no; *Age*: adolescents (16–19 years), youths (20–25 years) and young adults (26–29 years); *Education level*: compulsory education (≤ 9 years of formal school education), secondary education (10 to 12 years) and post-secondary education (> 12 years); *Economic stress*: yes/no, if during the last year they experienced difficulties in managing their regular expenses, for example, food, rent, bills, etc.; *Reason for migration*: to seek asylum, family reunion, work and other reasons of migration; *Religion*: Islam, Christianity and other beliefs, including atheism; *Need for interpreter*: yes/no, if they need language assistance when communicating with healthcare or other public services.

#### Stratification variables

The analysis was stratified based on the following variables: *Gender*: men, women, and non-binary (this category included also those who answered do not know or do not want to answer or reported that they had other gender identity/expression); *Sexual orientation*: heterosexual, LGBA (lesbian, gay, bisexual and asexual) and do not want to answer; *Region of birth*: Middle East and North Africa (MENA), South Asia (SA), sub-Saharan Africa (SSA) and “other”, including all countries that are not located in the previous three regions. These categories are based on the United Nations geographical regions; *Resident permit*: Waiting for a decision on residence permit in Sweden, had residence permit since 2016 or later, and had residence permit before 2016.

#### Sexual rights domains

In this study, we adapted the sexual rights definition according to the Guttmacher-Lancet Commission report [10]. The starting point of our survey was not based on the report’s definition, as our survey was designed before the report was published. However, the questions included in the survey reflect some key points of the Guttmacher-Lancet Commission’s definition of sexual rights. In order to operationalise this definition, we first grouped the eleven points included in that definition into five domains of sexual rights and then selected the questions from the survey that reflected these five domains (Table 1) (the full questions in each domain are attached in Additional file 1).

**Table 1:** Sexual rights’ domains

*Guttmacher-Lancet commission points*	*Domains explored in this study*	*Issues included in this domain*
1. Achieve the highest attainable standard of sexual health, including access to sexual and reproductive health services	**The right to the highest attainable sexual health and access to SRH services**	Self-rated sexual health, refraining from SRH services, perception of services and satisfaction with services
2. Seek, receive, and impart information related to sexuality 3. Receive comprehensive, evidence-based, sexuality education	**The right to access information and education about SRHR**	Ability to retrieve new information about SRHR and the ability to get contraceptives when needed
4. Have their bodily integrity respected	**The right to bodily integrity, free from coercion and violence**	Exposure to sexual violence
5. Choose their sexual partner 6. Decide whether to be sexually active or not 7. Engage in consensual sexual relations 8. Choose whether, when, and whom to marry 9. Enter into marriage with free and full consent and with equality between spouses in and at the dissolution of marriage 10. Make free, informed, and voluntary decisions about their sexuality, sexual orientation, and gender identity	**The right to make free, informed decisions about sexuality and sexual relations**	The ability to choose a sexual partner without restrictions.
11. Pursue a satisfying, safe, and pleasurable sexual life, free from stigma and discrimination	**The right to a satisfying and safe sexual life, free from stigma and discrimination**	Satisfaction with sexual life, the ability to have sex in a safe place and to suggest and use methods to avoid unwanted pregnancy, sexually transmitted diseases, and discrimination based on sexual identity and orientation

#### Analysis

Descriptive statistics were used to describe the sample characteristics and the frequencies of sexual rights’ fulfilment in different domains. Results were calculated for each category in gender, sexual orientation, residence permit and region of birth to assess the frequencies of fulfilment within subgroups of respondents. We used the software package Stata 15 for the analysis.

## Results

### Sample characteristics

Around two thirds of respondents identified themselves as men, one third as women and 2.0% as non-binary (Table 2). Approximately three fourths of respondents categorised themselves as heterosexual, 13% as lesbian, gay, bisexual or asexual (LGBA) and 14.2% did not want to answer this question. LGBA included, among others, homosexual (2.2%), bisexual (4.7%) and asexual (4.1%). More than half of respondents (57.4%) were born in the Middle East and North Africa (MENA), 18.2% in South Asia (SA), 21.5% in sub-Saharan Africa (SSA) and 3% in other parts of the world. Only one third of respondents had a residence permit in Sweden prior to 2016, while 11.6% were still waiting for a decision concerning their residence applications. More than half of respondents (55.8%) reported not having had sexual intercourse, and this was equally prevalent among men and women but less among non-binary people (45%). Respondents were almost equally distributed over the three age groups. Around one fourth of respondents had more than 12 years of formal education, and 44.9% had 9 years or less. Most respondents came to Sweden either to seek asylum (72.2%) or to reunify with a family member (20.4%). Two thirds of respondents were Muslims, while one fifth were Christians. Around half of respondents (52.8%) needed language assistance, sometimes when dealing with healthcare or other public services.

**Table 2:** Background characteristics of respondents

	n	%
**Gender**		
Woman	586	34.9
Man	1060	63.1
Non-binary	34	2.0
**Sexual orientation**		
Heterosexual	1134	73.1
LGBA	201	13.0
Do not want to answer	217	14.0
**Region of birth**		
Middle East & North Africa	989	57.4
South Asia	313	18.2
Sub-Saharan Africa	370	21.5
Other	51	3.0
**Residence permit**		
Waiting	188	11.6
2016 or later	911	56.0
Before 2016	528	32.5
**Ever had sexual intercourse**		
Yes	731	44.2
No	922	55.8
**Age**		
16–19 years	670	37.8
20–25 years	592	33.4
26–29 years	511	28.8
**Education level**		
≤ 9 years	751	44.9
10–12 years	482	28.8
> 12 years	441	26.3
**Economic stress**		
No	892	52.4
Yes	810	47.6
**Reason for migration**		
Asylum	1108	72.2
Family reunion	313	20.4
Work	46	3.0
Other	67	4.4
**Religion**		
Islam	1163	67.0
Christianity	339	19.5
Other	235	13.5
**Need for intrepreter**		
No	823	47.2
Yes	921	52.8

### The right to the highest standard of sexual health

**Fig. 2:**
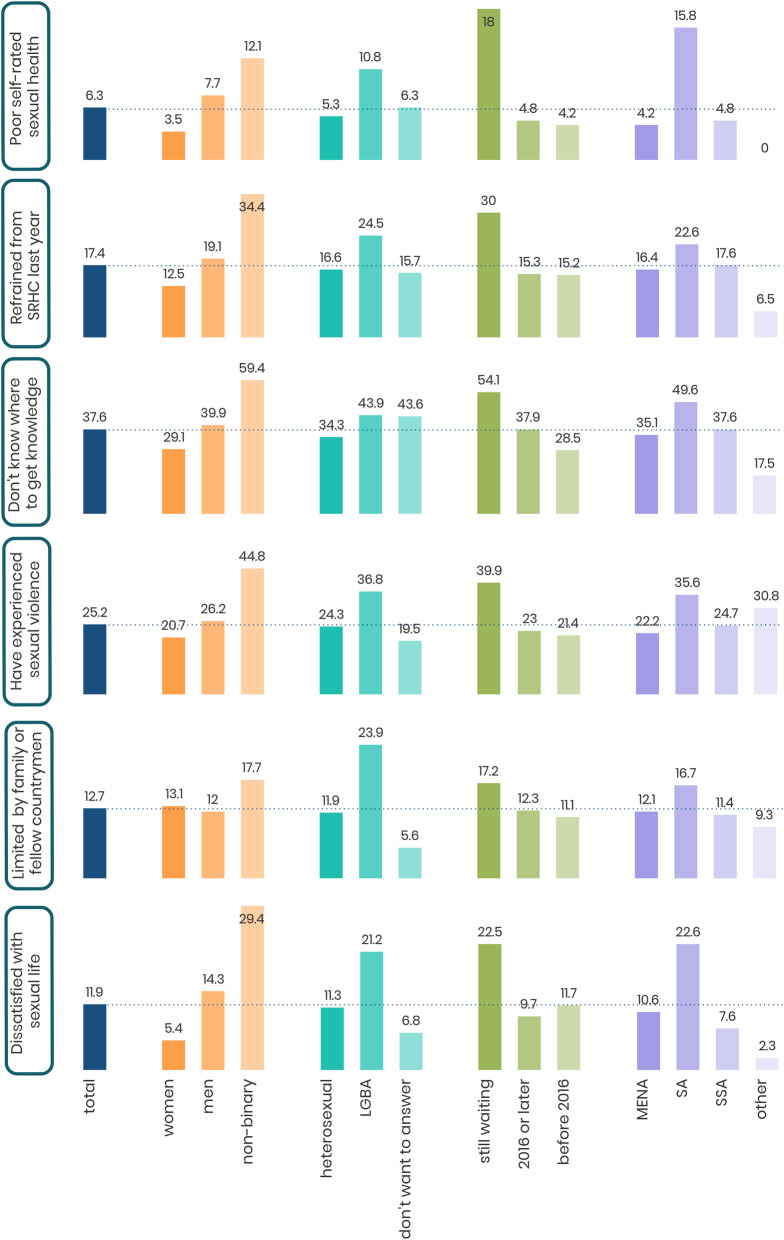
Illustration of selected indicators of sexual rights among young immigrants in Sweden. Stratified by gender, sexual orientation, residence permit and region of birth

Around three quarters (76.6%) of respondents perceived their sexual health as very good, good or fair (79.1% of men and 72.7% of women). In contrast, 6.3% perceived their sexual health as poor or very poor (7.7% of men and 3.5% of women). The highest proportion of those who perceived their sexual health as poor or very poor were found among non-binary individuals (12.1%), LGBA (10.8%), those waiting for a decision regarding residence permit (18.0%) and those born in South Asia (15.8%). (See Fig. 2 and Table 1 in Additional file 2 for more details).

### The right to access sexual and reproductive health services

About one sixth of respondents (17.4%) did not visit healthcare during the last year despite their felt needs of care (19.1% of men and 12.5% of women). The highest proportions of those who did not visit Healthcare despite felt needs were found among non-binary individuals (34.4%), those waiting for a residence permit (30.0%) and those born in South Asia (22.6%) (See Fig. 2). The most commonly reported reasons for not visiting healthcare despite felt needs were lack of knowledge about available health services (48%), long waiting times (20%), language difficulties (17%), financial difficulties (17%), lack of trust in the Swedish health system (11%) and a previous bad experience with the Swedish health services (10%). (See Table 2 in Additional file 2 for more details).

Of those who visited SRH services (13.9%), the majority agreed or strongly agreed that the services were good for these five dimensions: respect (92.6%), privacy (87.9%), non-judgement (71.6%), quality (75.4%) and equity (89.6%). In general, the highest proportion of those who perceived the services as bad in the aforementioned five dimensions of respect, privacy, non-judgement, quality and equity were found among men, non-binary individuals, those who did not want to answer about their sexual orientation, those waiting for a residence permit and those born in South Asia and sub-Saharan Africa (See Fig. 2).

Overall, 89.0% of those who visited SRH services were very satisfied, satisfied or neither satisfied nor dissatisfied with their visit. While, 11.0% reported to be either dissatisfied or very dissatisfied. The highest proportions of those who were dissatisfied or very dissatisfied were found among men (13.6%), those who did not want to answer about their sexual orientation (13.3%), those waiting for a residence permit (19.4%), and those born in South Asia (16.4%) (See Fig. 2).

### The right to access information and education related to SRHR

More than one third of respondents (37.6%) did not know where to get information about SRHR (29.1% of women and 39.9% of men). The highest proportions of respondents who lacked knowledge about where to get information were found among non-binary people (59.4%), LGBA (43.9%), those who did not want to answer about their sexual orientation (43.6%), those waiting for a residence permit (54.1%) and those born in South Asia (49.6%). (See Fig. 2 and Table 3 in Additional file 2 for more details).

Regarding information about where to get contraceptives, 53.4% of respondents answered that they knew where to get contraceptives, and 21.4% did not know. More women (60.8%) than men (49.4%) knew where to get contraceptives. The highest frequencies of those who did not know were found among non-binary individuals (40.0%), those who did not want to answer about their sexual orientation (30.9%), those waiting for a residence permit (34.0%) and those born in South Asia (30.6%) and sub-Saharan Africa (30.3%).

### The right to bodily integrity, free from coercion and violence

Around one quarter of the participants (25.2%) reported that they had been exposed to sexual violence (26.2% of men and 20.7% of women), and another 9.6% were not sure if it had happened (11.0% of men and 6.5% of women). The highest proportions were found among non-binary individuals (44.8%), LGBA (36.8%), those waiting for a residence permit (39.9%) and those born in South Asia (35.6%) followed by respondents from other regions of the world (30.8%). (Figure 2, see also Table 4 in Additional file 2 for more details).

The most common acts of sexual violence reported were sexual remarks (11.3%), exposing body parts (11.1%), touching sexual organs (10.0%), harassment through the internet (8.3%) and vaginal intercourse (6.5%).

Almost two thirds (63.6%) of those exposed to sexual violence refrained from reporting it to any person or authority (64.7% of men and 56.7% of women). The majority of those who reported the violence talked to a friend or a relative (29.0%) and only 1.9% reported to the police and social services, respectively.

### The right to make free, informed decisions about their sexuality and sexual relations

While nearly half of respondents (47.3%) did not feel limited, around four in ten respondents (37.5%) stated that they felt limited regarding with whom they can have an intimate relationship (36.0% of women and 37.3% of men). The highest proportions of those who felt limited were found among non-binary individuals (41.2%), LGBA (50.0%) and those born in sub-Saharan Africa (42.2%) (See Fig. 2). The grounds for feeling limited were either limiting oneself (22.7%), religious beliefs (13.5%), sexual identity (1.8%), sexual orientation (1.0%) or disability (0.8%) or limited by family or fellow countrymen (12.7%). The highest frequencies were among non-binary individuals (17.7%), LGBA (23.9%), those waiting for a residence permit (17.2%) and those born in South Asia (16.7%). (See Fig. 2 and Table 5 in Additional file 2 for more details).

### The right to a satisfying and safe sexual life, free from stigma and discrimination

Around two thirds (66.1%) of respondents were either very satisfied, satisfied, or neither satisfied nor dissatisfied with their sexual life (67.5% of men and 65.3%% of women), while 11.9% were dissatisfied or very dissatisfied (14.3% of men and 5.4% of women). The highest proportions of dissatisfaction were found among non-binary individuals (29.4%), LGBA (21.2%) those waiting for a residence permit (22.5%) and those born in South Asia (22.6%). (See Fig. 2, see also Table 6 in Additional file 2 for more details).

The majority (80.2%) of those who reported not having had sexual intercourse stated that they had their latest sexual encounter in a safe place (77.6% of men and 85.6% of women) while 10.1% stated that it was not (13.6% of men and 2.6% of women); 9.7% could not or did not want to answer. The highest proportions of those who did not have sex in a safe place were found among LGBA (14.8%), those waiting for a residence permit (17.0%) and those born in South Asia (14.8%) or sub-Saharan Africa (15.6%).

Of those who reported that they had sexual intercourse, 60.1% stated they could suggest using a condom or other contraceptives during their latest sexual encounter (61.0% of men and 59.8% of women). Nearly one in five (19.6%) stated that they could not. The highest proportions of those who could not were found among those waiting for a residence permit (26.4%) and those born in South Asia (27.3%).

During their latest sexual encounter, about four in ten respondents reported that they used a condom to protect themselves against unwanted pregnancies (42.4%) or HIV and other sexually transmitted infections (HIV/STIs) (35.7%). But, 4.3% stated they did not use any method to avoid pregnancy despite it being needed, while 7.8% stated that they did not use any method of protection against HIV/STIs despite it being needed. The highest proportions were found among men (10.3%), LGBA (11.6%), those waiting for a residence permit (12.3%) and those born in sub-Saharan Africa (11.7%).

Around one-third of the respondents (36.6%) mentioned that they had experienced discrimination in Sweden at least once during the previous year. The most reported grounds for discrimination were ethnicity or country of origin (62.9%) and religion (36.1%). About 4.7% stated that sexual orientation was the grounds for discrimination, and another 4.2% mentioned sexual identity. The highest proportions of discrimination based on sexual orientation and identity were found among non-binary and LGBA individuals; however, higher proportions of these two groups were being discriminated against based on other grounds for discrimination, e.g. ethnicity rather than sexual orientation or identity.

## Discussion

The results show that young migrants reported overall good sexual health, but some subgroups reported poor sexual health to a greater extent than others. In addition, there were considerable variations in the fulfilment of sexual rights between different domains and subgroups of respondents. Some subgroups of respondents such as men, non-binary, LGBA, South Asians and those who were still awaiting a decision for residence permit reported worse outcomes to a greater extent than other groups in almost all domains. This study suggests that migrants are a heterogenous group consisting of people with different socio-economic backgrounds, which might affect their vulnerabilities and opportunities to enjoy their right to the highest level of sexual health attainable.

### The right to good sexual health

Most respondents reported enjoying good sexual health. This result is in line with the international literature on the healthy migrant effect, which suggests that newly arrived migrants are often healthier than host populations but that their health status deteriorates with their length of stay due to complex factors linked to migration [27–29]. However, non-binary individuals, LGBA, South Asians and those who were still awaiting a decision reported poor sexual health to a greater extent than other groups. Our results corroborate other studies and national surveys that show almost similar patterns among people with non-normative gender identity and sexual orientation [24, 25]. This suggests that LGBA are at an increased risk of poor health outcomes [5, 30] and being a migrant can exacerbate the situation as it might put them in vulnerable intersections (young age, migrant, unknown legal status, non-binary, LGBA and so on) [30].

Other vulnerable subgroups identified in this study are respondents from South Asia and those who were still awaiting a decision about their residence permit. Respondents from South Asia were overrepresented among those who were still awaiting a decision and were mainly comprised of boys and young men from Afghanistan, a country whence a high proportion of vulnerable unaccompanied children and young people living in Sweden come. This may be an indication that unaccompanied boys and young men from Afghanistan are one of the most vulnerable subgroups of migrants. Other studies have also shown that unaccompanied or orphaned minors constitute one of the most vulnerable subgroups of migrants who suffer disproportionately poor health [2, 16]. Moreover, people living with an unknown legal status in host countries are also often described in the literature as one of the most vulnerable subgroups of migrants. They often live in limbo with limited legal and social rights, separated from their partners and families [29, 31]. These multiple and complex issues can generate complex health and social needs and increase vulnerability to poor sexual health.

### The right to access SRH information and services

The results further show that nearly one in five (17%) respondents stated that they refrained from seeking care despite perceived needs, which may indicate that they were not fully enjoying the right to access services. Similar results were reported in a study of knowledge and utilisation of SRH services among Thai women living in Sweden that also found that most participants had not sought care despite expressed needs [32]. Other studies have shown that foreign born persons refrain from seeking care twice as often as Swedish-born [33]. The existing literature has shown that migrants often face complex barriers that limit their access to available services and information in host countries [21, 34]. Lack of knowledge about available services (where to go) was the most cited reason for not seeking care among participants in this study, suggesting that their right to access information was not being fulfilled to a greater extent. Similar results were found in other studies of knowledge and use of SRH services among immigrant women in Sweden [32, 35]. Other reasons cited for refraining from seeking care were long waiting times, not being able to afford care, distrust of the Swedish health system, difficulties in reaching healthcare on the telephone, negative experiences from previous encounters/visits and language barriers, which is consistent with existing literature [21, 34]. The fact that more than half of the respondents in this study needed interpreters during medical encounters might be an indication that readily available information can actually be inaccessible to young migrants due to language barriers.

However, our results also show disparities in the fulfilment of the right to access information and services. For instance, LGBA and non-binary individuals, respondents from South Asia and those who were still waiting for a decision regarding residence permit reported refraining from seeking care to a greater extent than other groups. Other studies have also found that LGBA and non-binary people are more likely to report unfavourable experiences when accessing services and avoid seeking care, including emergency care because of stigmatisation, discrimination and bias that they experience in many healthcare settings [22, 30]. Those who were still awaiting a decision regarding residence permit might refrain from seeking available services due to legal barrier that limit their access or fear of deportation [21, 34]. Nevertheless, the majority of those who reported that they used SRH-related services during the previous year claimed that they were treated with respect, privacy, and without prejudice or discrimination and were satisfied with the care provided.

### The right to make a free decision about sexuality and sexual relations

Sexual rights guarantee all people the right to choose their partner without coercion, discrimination or violence regardless of gender, gender identity, sexual orientation, religion, age, ethnicity or disability. Our results show that four in ten respondents did not enjoy this right, and the proportion of respondents who reported enjoying it was low (47%) compared to the 87% reported in the FOKUS 15 survey on sexual and reproductive rights among the general youth (16–25 years) population in Sweden [26]. In addition, the FOKUS 15 survey showed that young people with a foreign background (69%) and who were non-heterosexual (74%) reported enjoying the right to freely choose their partner to a lesser extent than young native Swedes (93%) and heterosexual youth (90%) [26]. Even if respondents in our study reported to the greatest extent limiting themselves in their choice of intimate partner, it is also evident that religion, family members, and fellow countrymen played a role in infringing on young migrants’ right to freely choose their intimate partners. These restrictions constitute a violation of their sexual rights and collide with internationally recognised human rights and national legislation [10, 12]. Such restrictions were reported to a greater extent among non-binary people, LGBA, respondents from South Asia and those who were still waiting for a decision regarding residence permit; this may increase the risk for early and forced marriage as well as honour-related violence and oppression [3, 36].

### The right to be free from coercion and violence

Sexual rights also guarantee all people the right to decide on their body regarding sexuality, which includes, among other things, the right to say yes or no to sex. This study shows that a quarter of respondents were victims of different types of sexual violence and harassment, which was similar to the proportion reported in this age group (24 respective 26%) in the SRHR 2017 survey of SRHR in the general population [25], but lower than the number in the UngKAB15 survey [24] and in the Keygnaert et al. study on sexual and gender-based violence in refugees, applicants, and undocumented migrants in Belgium and the Netherlands [37]. However, contrary to what has been reported in previous research [37–39] and in the national SRHR 2017 and UngKAB15 surveys [24, 25], young migrant men were more likely to report sexual violence compared to young migrant women in this study, but again the highest proportions were found among non-binary people, LGBA, those waiting for a residence permit and those born in South Asia. The vulnerability of men to sexual violence has also been stressed in previous research [38] and in a recent report on sexual violence against men and boys in Syria that indicates that while women and children carry the major burden of sexual violence, men and transgender individuals, must also be taken into consideration [40]. A meta-analysis of studies focussing on lesbian, gay and bisexual (LGB) people indicated that up to 55% of them experienced verbal harassment, 45% experienced sexual harassment and 41% experienced higher levels of discrimination than the general population [41].

Sexual abuse and exploitation of unaccompanied boys and young men from South Asia and the Middle East by perpetrators from their own and host communities in Europe has been well documented in Greece, Italy and Sweden, underlining post-migration vulnerability in host countries [16, 17]. Yet, sexual violence can take place during the journey and before migration, particularly in conflict settings [39]. For instance, sexual abuse and exploitation of boys and young men are common in Afghanistan through the so-called “Bacha Bazi” or “dancing boys” phenomenon that is prevalent but underreported [42]. Apart from the ongoing conflict, this may be one of the reasons that forced many young Afghan boys to flee their country. Moreover, a recent report from the United Nations High Commissioner for Refugees showed that men and boys are subjected to sexual violence, including sexual torture, by different groups involved in the conflict in Syria [40].

Additionally, those who were still waiting for a decision regarding residence permit might be in a more vulnerable situation with limited legal and social rights and thus are at increased risk of sexual exploitation and violence. At the same time, various barriers reported in this study can impede both access to and delivery of service to migrant victims of sexual violence in general, and particularly to male victims. This may, in addition to other barriers, (partly) explain why most victims in this study did not disclose or report past or recent sexual violence.

### The right to a satisfying and safe sexual life, free from stigma and discrimination

Most respondents were satisfied with their sexual life, but young men reported being dissatisfied with their sexual life to a greater extent than women. Similar patterns have been found in the SRHR2017, Youth and sexuality Barometer 2014 and UngKAB15 surveys [23–25], emphasising the need for more studies on young men’s sexuality and sexual satisfaction. Most respondents (80%) also declared that their latest sexual encounter took place in a safe place, but this proportion was lower than what was reported in the UngKAB15 (95%) [24], suggesting that young migrants may have reduced access to safe places to have sex. Moreover, contrary to the UngKAB15 survey that found no gender differences [24], the proportion of young men who did not have their latest sexual encounter in a safe place was almost five times that of young women. About four in ten respondents claimed that they used a condom during their latest sexual encounter to protect themselves against unwanted pregnancies (42%) or HIV/STI (36%), which is higher than the 25% reported in other national surveys [23, 24]. However, the proportion of respondents who reported that they could suggest using a condom or other contraceptives during their latest sexual encounter in this study was lower than the 89% reported in the UngKAB15 survey [24], suggesting that young migrants are in a more vulnerable position in sexual relationships than the young population in general.

Around 37% of the respondents reported being discriminated against in Sweden the previous year, which was higher than the 22% and 29% reported in the UngKAB15 and the Youth and sexuality Barometer 2014 surveys respectively. But, the most cited reasons for discrimination in Sweden was ethnicity and religion, not sexual orientation or gender identity. Other national surveys also showed that sexual orientation and identity were not the most common grounds for discrimination among young people in Sweden, but the proportion of discrimination based on sexual orientation in national surveys was higher (almost double) than what is reported in this study [23, 24]. LGBA and non-binary migrants may anticipate discrimination and thus conceal their sexual orientation and identity. In addition, the fact that LGBA and non-binary respondents in this study represented the highest proportion of those who reported being discriminated because of their sexual orientation or identity suggests that they might be simultaneously discriminated against on multiple grounds. Our results are in line with other studies that found that people who have non-normative gender identity or sexual orientation often face social exclusion, marginalisation and discrimination [5, 30], suggesting that there are still significant obstacles to full recognition and enjoyment of non-binary and non-heterosexual people’s fundamental rights despite legislation protecting against discrimination based on these grounds in Sweden.

### Limitations and strengths

The descriptive design, convenience sampling and self-report nature of the study and the unknown validity of the questionnaire may threaten validity and make the results difficult to generalise to other groups of migrants as the findings may only reflect the situation among newly arrived migrants. Additionally, social desirability could have influenced participants to report less violations of sexual rights in Sweden. However those who had no residence permit did reported more violations compared to other groups suggesting a limited role for social desirability.

Even though, we think that the characteristics of the sample in regards to gender and region of birth reflects the structure of newly arrived immigrants to Sweden, the sample is not representative when considering other characteristics. However, our sampling strategy was guided by a participatory approach and the need to include and reflect a heterogeneous migrant group through the combination of “location” and “snowball” samplings, which have been documented as appropriate in previous migrant research [43]. The survey was also administered in various ways in order to reach different sociodemographic groups of migrants, however, the different data collection methods might have led to different results because of the way participants answered some sensitive questions.

The questionnaire was translated into the five most-spoken languages of the target population to reach a lot of respondents, and back translation was used to check for accuracy. In cases when the respondents had a limited literacy (less than 1% of respondents), they were assisted by bicultural team members during data collection in schools. Moreover, using similar questions to national surveys allowed for comparison and to highlight the young migrants’ situation and the vulnerability of certain subgroups. Finally, the operationalisation of different dimensions of the Guttmacher-Lancet commission definition of sexual rights into measurable variables provides unique insights in this study. Future research should adopt an analytical approach to identify factors affecting sexual health and rights among young migrants.

## Conclusions

The findings revealed gender inequities in the fulfilment of sexual rights as well as considerable variations between different domains. Although, the majority of individuals reported overall good sexual health, the rights to access services and information about SRHR, to make free decisions about sexuality and sexual relations, to be free from coercion and violence, to have a satisfying sexual life free from stigma and discrimination were somewhat fulfilled but not equally for all groups as some subgroups reported not enjoying it to a larger extent than others. Men, non-binary individuals, LGBA, South Asians and those who were still awaiting a decision regarding residence permit appeared to be the most vulnerable subgroups. To improve awareness about sexual rights and access to available services on equal terms, timely and culturally adapted information about sexual rights, gender equalities, laws protecting sexual rights and available services in Sweden should be provided in appropriate languages and formats. Tailored attention should be paid to specific vulnerable subgroups.

## Supplementary Information

**Additional file 1.** Full list of questions in the five domains of sexual rights. Supplementary information of To what extent are young migrants’ sexual rights being fulfilled in Sweden? A cross-sectional study. Mazen Baroudi, Anna-Karin Hurtig, Isabel Goicolea, Miguel San Sebastian, Robert Jonzon, Faustine Kyungu Nkulu-Kalengayi.

**Additional file 2.** Detailed results of all questions in the five domains of sexual rights. Supplementary information of To what extent are young migrants’ sexual rights being fulfilled in Sweden? A cross-sectional study. Mazen Baroudi, Anna-Karin Hurtig, Isabel Goicolea, Miguel San Sebastian, Robert Jonzon, Faustine Kyungu Nkulu-Kalengayi.

## Data Availability

The datasets used and/or analysed during the current study are available from the corresponding author on reasonable request.

## Abbreviations

HRV: Honour-related violence
LGBQA: Lesbian, gays, bisexual, queer or asexual
LGBTI: Lesbian, gay, bisexual, trans and intersex
MENA: Middle East and North Africa
SA: South Asia
SRH: Sexual and reproductive health
SRHR: Sexual and reproductive health and rights
SSA: Sub-Saharan Africa
WHO: World Health Organisation
