# Supplementary material for: Young migrants’ sexual rights in Sweden: a cross-sectional study
Source: BMC Public Health. 2021 Sep 6;21:1618. doi: 10.1186/s12889-021-11672-1 (PMC8420038; doi:10.1186/s12889-021-11672-1)
Supplement: Supplementary file 1 — Additional file 1. Full list of questions in the five domains of sexual rights. Supplementary information of To what extent are young migrants’ sexual rights being fulfilled in Sweden? A cross-sectional study. Mazen Baroudi, Anna-Karin Hurtig, Isabel Goicolea, Miguel San Sebastian, Robert Jonzon, Faustine Kyungu Nkulu-Kalengayi. [file 12889_2021_11672_MOESM1_ESM.pdf]

Additional file 1: Full list of questions in the five domains of sexual rights.

The right to the highest standard of sexual health

1. How would you rate your sexual health?
   - ☐ Very good
   - ☐ Good
   - ☐ Fair
   - ☐ Poor
   - ☐ Very poor
   - ☐ Don't know

The right to access sexual and reproductive health services

2. Have you in the last 12 months visited healthcare service/social services regarding sexual or reproductive health issues?
   - ☐ No
   - ☐ Yes
   - ☐ Don't know

3. During your last visit, how much do you agree with the following statements?

	Strongly agree	Agree	Disagree	Strongly disagree
a. I felt that I was treated with respect	☐	☐	☐	☐
b. The conversation or the examination happened in a way that no one else can see or hear us	☐	☐	☐	☐
c. The staff had an unprejudiced attitude towards me	☐	☐	☐	☐
d. I received treatment or help that met my expectations	☐	☐	☐	☐
e. I felt treated fairly without discrimination or offense	☐	☐	☐	☐

4. How satisfied were you with the service you received in your last visit?
   - ☐ Very satisfied
   - ☐ Satisfied
   - ☐ Neither satisfied nor dissatisfied
   - ☐ Somewhat dissatisfied
   - ☐ Very dissatisfied

5. In the previous 12 months have you felt that you need sexual or reproductive healthcare but did not seek care?
   - ☐ No
   - ☐ Yes

The right to access information and education related to SRHR

6. If you need more knowledge, do you know where to get this knowledge?
   - ☐ Yes
   - ☐ No

7. If you need contraceptives, do you know where to get them?
   - ☐ Yes
   - ☐ No
   - ☐ Not applicable

The right to bodily integrity, free from coercion and violence

8. Have you ever experienced any of the following acts **against your will**?

	Yes	No, it did not happen	Don't know/ Not sure
a. Harassments with sexual words or remarks.	☐	☐	☐
b. Someone exposed him-/herself indecently in front of me	☐	☐	☐
c. Someone touched my genitals or breasts	☐	☐	☐
d. I had to masturbate for someone	☐	☐	☐
e. I had vaginal sexual intercourse	☐	☐	☐
f. I had oral sex	☐	☐	☐
g. I had anal sexual intercourse	☐	☐	☐
h. Sexually harassment through internet or social media	☐	☐	☐
i. Someone distributed nude photographs of you on the Internet	☐	☐	☐
j. Other:	☐	☐	☐

☐ No, I have never been subject to any of the above → Go to question 52

9. Was the person in the previous question:
   You can select several options.
   - ☐ Someone you have, or have been, in a relationship with (partner)
   - ☐ A family member or a friend
   - ☐ A teacher, boss, employer or a, supervisor
   - ☐ A colleague or a classmate
   - ☐ Someone I didn't know (a stranger)
   - ☐ Other: ____________

10. Did you talk or report to anyone about this?
You can select several options.
- ☐ Yes, I talked to a friend or a relative
- ☐ Yes, I talked to a teacher/school counsellor
- ☐ Yes, I informed my boss/employer
- ☐ Yes, I informed the social services (socialtjänst)
- ☐ Yes, I reported to the police
- ☐ Other, please specify
- ☐ No

The right to make free, informed decisions about their sexuality and sexual relations

11. Do you feel yourself limited by your family or your immediate surroundings in terms of whom you can have an intimate relationship with?
You can mark more than one alternative.
- ☐ No
- ☐ No, not by the others but I limit myself
- ☐ Yes, by my family/ parents/siblings
- ☐ Yes, by my country fellows
- ☐ Yes, because of my religious beliefs
- ☐ Yes, because of my sexual identity
- ☐ Yes, because of my sexual orientation
- ☐ Yes, because of a disability
- ☐ I miss a response alternative that suits me
- ☐ Don't know

The right to a satisfying and safe sexual life, free from stigma and discrimination

12. How satisfied are you with your current sex life?
- ☐ Very satisfied
- ☐ Satisfied
- ☐ Neither satisfied nor dissatisfied
- ☐ Dissatisfied
- ☐ Very dissatisfied
- ☐ Not applicable

13. If you think about the last time you had sex, do you agree with the following claims?

	No	Yes	Can't answer/ don't want to answer
a. I had sex in a safe place where I felt secure	☐	☐	☐
b. I felt that I could suggest and use a condom or other contraceptive if I wanted to	☐	☐	☐

14. Did you/your partner used any of the following **to avoid pregnancy** during the most recent sexual encounter?

    You can select several options.

    ☐ Yes, contraceptive pills or other hormonal method
    ☐ Yes, a condom
    ☐ Yes, intrauterine device
    ☐ Yes, a natural family planning (safe periods and/or withdrawal)
    ☐ Yes, emergency contraceptive pills

    ☐ No, but it was needed
    ☐ No, because we were planning to have a child
    ☐ No, I don't know how to do it or what to do
    ☐ No, because we had sex in a way which did not necessitate condoms or other contraceptives

    ☐ I'm unsure/don't know
    ☐ Other: ______

15. Did you/your partner **protect yourselves from sexually transmitted diseases** during the most recent sexual encounter?

    You can select several options.

    ☐ Yes, a condom

    ☐ No, I don't think it is important
    ☐ No, but it was needed
    ☐ No, as we were planning to have a child
    ☐ No, I don't know how to do it or what to do
    ☐ No, as we had sex in a way that did not necessitate any protection

    ☐ I'm unsure/don't know
    ☐ Other: ______

16. During the last 12 months in Sweden, have you been treated/addressed in a way that made you feel discriminated against or offended?

    ☐ No
    ☐ Yes, once
    ☐ Yes, several times

17. Was the discriminatory or offensive treatment associated with any of the following?
    You can select several options.
    - ☐ Ethnic origin or country of origin
    - ☐ Sex (that you were born as a male or a female)
    - ☐ Sexual orientation (whether you feel attracted to or prefer to have sex with people of same, opposite sex or both)
    - ☐ Age
    - ☐ Disability
    - ☐ Religion
    - ☐ Sexual identity (how you identify yourself as a man, woman…)
    - ☐ Other, please specify: ________
    - ☐ Don't know
