# Supplementary material for: Young migrants’ sexual rights in Sweden: a cross-sectional study
Source: BMC Public Health. 2021 Sep 6;21:1618. doi: 10.1186/s12889-021-11672-1 (PMC8420038; doi:10.1186/s12889-021-11672-1)
Supplement: Supplementary file 2 — Additional file 2. Detailed results of all questions in the five domains of sexual rights. Supplementary information of To what extent are young migrants’ sexual rights being fulfilled in Sweden? A cross-sectional study. Mazen Baroudi, Anna-Karin Hurtig, Isabel Goicolea, Miguel San Sebastian, Robert Jonzon, Faustine Kyungu Nkulu-Kalengayi. [file 12889_2021_11672_MOESM2_ESM.pdf]

To what extent are young migrants' sexual rights being fulfilled in Sweden? A cross-sectional study.

Baroudi, M. Hurtig, A-K. Goicolea, I. San Sebastian, M. Jonzon, R. Nkulu-Kalengayi, F

Additional file 2: Detailed results of all questions in the five domains of sexual rights.

Table1: The right to the highest attainable sexual health

	Women	Men	non-binary	Heterosexu	LGBA	Don't want	Still waiting	2016 or lat	before 201	MENA	SA	SSA	Other	Total
	n(%)	n(%)	n(%)	n(%)	n(%)	n(%)	n(%)	n(%)	n(%)	n(%)	n(%)	n(%)	n(%)	n(%)
**Self-rated sexual health**														
good/fair	399(72,7)	788(79,1)	22(66,7)	886(80)	143(73,7)	129(62)	107(64,1)	685(78,8)	387(77,9)	729(76,6)	188(66)	283(84,7)	33(71,7)	1230(76,3)
poor	19(3,5)	77(7,7)	4(12,1)	59(5,3)	21(10,8)	13(6,3)	30(18)	42(4,8)	21(4,2)	40(4,2)	45(15,8)	16(4,8)	0(0)	101(6,3)
don't know	131(23,9)	131(13,2)	7(21,2)	162(14,6)	30(15,5)	66(31,7)	30(18)	142(16,3)	89(17,9)	183(19,2)	52(18,3)	35(10,5)	13(28,3)	283(17,5)

Table2: The right to access sexual and reproductive health services

	Women	Men	non-binar	Heterosexu	LGBA	Don't want	Still waiting	2016 or lat	before 201	MENA	SA	SSA	Other	Total
	n(%)	n(%)	n(%)	n(%)	n(%)	n(%)	n(%)	n(%)	n(%)	n(%)	n(%)	n(%)	n(%)	n(%)
**Visited SRH services**														
no	423(75,5)	849(84,4)	24(80)	911(83)	145(74)	174(82,9)	138(78)	691(80,2)	424(83,6)	794(83,6)	227(78,3)	274(79)	38(79,2)	1330(81,5)
yes	124(22,1)	101(10)	4(13,3)	152(13,8)	40(20,4)	20(9,5)	22(12,4)	132(15,3)	68(13,4)	134(14,1)	39(13,5)	48(13,8)	7(14,6)	228(13,9)
don't know	13(2,3)	56(5,6)	2(6,7)	35(3,2)	11(5,6)	16(7,6)	17(9,6)	39(4,5)	15(3)	22(2,3)	24(8,3)	25(7,2)	3(6,3)	74(4,5)
**Treated with respect**														
agree	108(93,1)	100(90,9)	5(83,3)	140(95,2)	39(90,7)	21(77,8)	23(79,3)	61(93,9)	127(94,1)	135(95,7)	37(90,2)	34(85)	8(88,9)	214(92,6)
disagree	8(6,9)	10(9,1)	1(16,7)	7(4,8)	4(9,3)	6(22,2)	6(20,7)	4(6,2)	8(5,9)	6(4,3)	4(9,8)	6(15)	1(11,1)	17(7,4)
**Privacy ensured**														
agree	98(90,7)	76(85,4)	2(50)	120(89,6)	26(81,3)	19(86,4)	14(66,7)	52(88,1)	109(92,4)	116(94,3)	22(73,3)	29(78,4)	7(87,5)	174(87,9)
disagree	10(9,3)	13(14,6)	2(50)	14(10,5)	6(18,8)	3(13,6)	7(33,3)	7(11,9)	9(7,6)	7(5,7)	8(26,7)	8(21,6)	1(12,5)	24(12,1)
**No-judgement from staff**														
agree	76(75,3)	49(67,1)	2(40)	88(74,6)	17(63)	13(59,1)	8(40)	36(66,7)	82(78,9)	92(83,6)	13(48,2)	14(45,2)	7(87,5)	126(71,6)
disagree	25(24,8)	24(32,9)	3(60)	30(25,4)	10(37)	9(40,9)	12(60)	18(33,3)	22(21,2)	18(16,4)	14(51,9)	17(54,8)	1(12,5)	50(28,4)
**Received expected help**														
agree	87(79,8)	58(69,9)	1(20)	101(75,9)	22(81,5)	13(56,5)	11(55)	47(75,8)	86(77,5)	94(76,4)	19(70,4)	24(72,7)	7(87,5)	144(75,4)
disagree	22(20,2)	25(30,1)	4(80)	32(24,1)	5(18,5)	10(43,5)	9(45)	15(24,2)	25(22,5)	29(23,6)	8(29,6)	9(27,3)	1(12,5)	47(24,6)
**No discrimination**														
agree	102(90,3)	73(86,9)	3(60)	120(88,9)	26(89,7)	19(82,6)	13(72,2)	54(87,1)	105(90,5)	114(92,7)	26(86,7)	33(82,5)	7(87,5)	180(89,6)
disagree	11(9,7)	11(13,1)	2(40)	15(11,1)	3(10,3)	4(17,4)	5(27,8)	8(12,9)	11(9,5)	9(7,3)	4(13,3)	7(17,5)	1(12,5)	21(10,5)
**Satisfied with the services**														
satisfied/ok	122(91,7)	114(86,4)	6(100)	155(89,6)	43(89,6)	26(86,7)	25(80,7)	73(93,6)	140(89,2)	137(89,5)	46(83,6)	51(91,1)	9(100)	243(89)
dissatisfied	11(8,3)	18(13,6)	0(0)	18(10,4)	5(10,4)	4(13,3)	6(19,4)	5(6,4)	17(10,8)	16(10,5)	9(16,4)	5(8,9)	0(0)	30(11)
**Refrained from SRH services**														
No	478(87,6)	790(80,9)	21(65,6)	899(83,4)	148(75,5)	172(84,3)	119(70)	714(84,7)	423(84,8)	788(83,7)	213(77,5)	276(82,4)	43(93,5)	1320(82,6)
Yes	68(12,5)	186(19,1)	11(34,4)	179(16,6)	48(24,5)	32(15,7)	51(30)	129(15,3)	76(15,2)	154(16,4)	62(22,6)	59(17,6)	3(6,5)	278(17,4)

Table3: The right to access information and education about SRHR

	Women	Men	non-binary	Heterosexu	LGBA	Don't want	Still waitin	2016 or lat	before 201	MENA	SA	SSA	Other	Total
	n(%)	n(%)	n(%)	n(%)	n(%)	n(%)	n(%)	n(%)	n(%)	n(%)	n(%)	n(%)	n(%)	n(%)
**Know where to get more knowledge**														
yes	376(70,9)	561(60,1)	13(40,6)	690(65,7)	105(56,2)	106(56,4)	73(45,9)	499(62,1)	347(71,6)	597(64,9)	128(50,4)	199(62,4)	33(82,5)	957(62,4)
no	154(29,1)	372(39,9)	19(59,4)	360(34,3)	82(43,9)	82(43,6)	86(54,1)	304(37,9)	138(28,5)	323(35,1)	126(49,6)	120(37,6)	7(17,5)	576(37,6)
**Know where to get contraceptives**														
yes	329(60,8)	461(49,4)	11(36,7)	614(57,7)	98(53)	67(34,5)	76(49,7)	422(51,5)	275(56,6)	463(49,9)	148(57,4)	182(58)	30(73,2)	823(53,4)
no	101(18,7)	207(22,2)	12(40)	199(18,7)	44(23,8)	60(30,9)	52(34)	161(19,7)	89(18,3)	152(16,4)	79(30,6)	95(30,3)	4(9,8)	330(21,4)
not applicable	111(20,5)	265(28,4)	7(23,3)	252(23,7)	43(23,2)	67(34,5)	25(16,3)	236(28,8)	122(25,1)	313(33,7)	31(12)	37(11,8)	7(17,1)	388(25,2)

Table4: The right to be free from coercion and violence

	Women	Men	non-binary	Heterosexu	LGBA	Don't want	Still waiting	2016 or lat	before 201	MENA	SA	SSA	Other	Total
	n(%)	n(%)	n(%)	n(%)	n(%)	n(%)	n(%)	n(%)	n(%)	n(%)	n(%)	n(%)	n(%)	n(%)
**Ever experienced sexual harassment**														
no	391(72,8)	594(62,8)	11(37,9)	708(66,5)	99(53,5)	134(67)	62(38)	343(69,9)	572(70)	669(70,9)	122(46,2)	202(64,7)	23(59)	1016(65,2)
yes	111(20,7)	248(26,2)	13(44,8)	258(24,3)	68(36,8)	39(19,5)	65(39,9)	105(21,4)	188(23)	209(22,2)	94(35,6)	77(24,7)	12(30,8)	392(25,2)
Don't know/not	35(6,5)	104(11)	5(17,2)	98(9,2)	18(9,7)	27(13,5)	36(22,1)	43(8,8)	57(7)	65(6,9)	48(18,2)	33(10,6)	4(10,3)	150(9,6)
**which type of harrassment**														
sexual remarks	74(14,2)	74(8,5)	7(25,9)	102(10,1)	38(23,5)	15(7,9)	31(21,5)	80(10,4)	41(8,7)	96(10,6)	39(16,4)	26(9,6)	4(10,3)	165(11,3)
exposing body	34(6,9)	100(12,5)	7(29,2)	93(9,9)	30(22,1)	16(9,1)	25(21,9)	80(10,8)	38(8,7)	93(10,6)	30(15,4)	23(9,8)	3(7,9)	149(11,1)
touch	42(8,6)	73(9,2)	5(21,7)	75(8)	30(22,4)	16(9,4)	17(15)	66(9)	38(8,9)	79(9,1)	24(12,8)	29(12,2)	2(5,4)	134(10)
masturbate	11(2,3)	27(3,5)	4(18,2)	26(2,8)	7(5,3)	9(5,3)	14(12,7)	20(2,8)	9(2,1)	22(2,6)	14(7,5)	6(2,6)	2(5,3)	44(3,4)
vaginal sex	22(4,5)	57(7,3)	2(9,1)	61(6,6)	12(8,8)	8(4,7)	15(13,6)	35(4,8)	28(6,5)	25(2,9)	17(9,1)	37(16)	6(16,2)	85(6,5)
oral sex	16(3,3)	52(6,5)	5(22,7)	46(4,9)	13(9,6)	12(6,9)	13(11,4)	38(5,2)	16(3,7)	33(3,8)	21(10,9)	15(6,3)	3(7,9)	72(5,4)
ananl sex	13(2,7)	26(3,3)	5(25)	21(2,3)	15(11)	7(4,2)	11(10)	17(2,3)	13(3)	22(2,5)	13(7,1)	6(2,6)	3(8,3)	44(3,4)
through interne	35(7,2)	61(7,8)	8(34,8)	71(7,6)	21(15,6)	10(5,9)	16(14,3)	52(7,1)	27(6,3)	73(8,4)	19(10,3)	15(6,4)	3(8,3)	110(8,3)
nude photos	4(0,8)	15(1,9)	2(8,7)	13(1,4)	3(2,3)	4(2,4)	5(4,6)	9(1,3)	7(1,7)	15(1,7)	2(1,1)	3(1,3)	2(5,6)	22(1,7)
other	10(2,3)	19(2,7)	1(5,6)	19(2,3)	5(4,3)	4(2,5)	7(6,9)	11(1,7)	9(2,3)	22(2,8)	6(3,8)	5(2,2)	0(0)	33(2,8)
**who was the perpetrator**														
partner	28(31,1)	42(22,5)	2(18,2)	47(24,5)	19(29,2)	7(25,9)	13(22)	29(21,2)	25(30,1)	32(21,1)	16(20)	24(37,5)	3(37,5)	75(24,7)
family/friend	14(15,6)	30(16)	2(18,2)	25(13)	12(18,5)	6(22,2)	10(17)	19(13,9)	12(14,5)	25(16,5)	12(15)	10(15,6)	1(12,5)	48(15,8)
teacher/boss	4(4,4)	6(3,2)	0(0)	7(3,7)	1(1,5)	1(3,7)	2(3,4)	2(1,5)	5(6)	7(4,6)	2(2,5)	0(0)	1(12,5)	10(3,3)
collegue	9(10)	35(18,7)	3(27,3)	31(16,2)	9(13,9)	5(18,5)	8(13,6)	24(17,5)	12(14,5)	22(14,5)	11(13,8)	12(18,8)	0(0)	45(14,8)
stranger	48(53,3)	82(43,9)	5(45,5)	95(49,5)	28(43,1)	11(40,7)	27(45,8)	73(53,3)	34(41)	76(50)	45(56,3)	18(28,1)	3(37,5)	142(46,7)
other	4(4,4)	13(7)	2(18,2)	12(6,3)	7(10,8)	2(7,4)	6(10,2)	6(4,4)	9(10,8)	13(8,6)	6(7,5)	4(6,3)	1(12,5)	24(7,9)
**Did you talk/report about it**														
yes friend/relati	34(37,8)	56(27,5)	2(16,7)	65(31,9)	17(25,8)	6(18,8)	12(19,4)	49(33,3)	29(34,5)	50(30,9)	20(23,3)	19(30,2)	4(40)	93(29)
yes teacher/cou	3(3,3)	10(4,9)	0(0)	7(3,4)	5(7,6)	1(3,1)	4(6,5)	6(4,1)	3(3,6)	7(4,3)	7(8,1)	0(0)	0(0)	14(4,4)
yes boss/employ	1(1,1)	2(1)	0(0)	2(1)	1(1,5)	0(0)	1(1,6)	2(1,4)	0(0)	1(0,6)	1(1,2)	1(1,6)	0(0)	3(0,9)
yes social servic	2(2,2)	4(2)	0(0)	4(2)	2(3)	0(0)	1(1,6)	1(0,7)	3(3,6)	2(1,2)	2(2,3)	2(3,2)	0(0)	6(1,9)
yes police	3(3,3)	3(1,5)	0(0)	4(2)	1(1,5)	0(0)	0(0)	3(2)	2(2,4)	3(1,9)	3(3,5)	0(0)	0(0)	6(1,9)
no	51(56,7)	132(64,7)	9(75)	127(62,3)	42(63,6)	23(71,9)	43(69,4)	88(59,9)	51(60,7)	103(63,6)	55(64)	40(63,5)	6(60)	204(63,6)
other	5(5,6)	7(3,4)	1(8,3)	8(3,9)	3(4,6)	2(6,3)	3(4,8)	6(4,1)	5(6)	9(5,6)	4(4,7)	2(3,2)	0(0)	15(4,7)

Table5: The right to make free informed decision about sexuality and sexual relations

	Women	Men	non-binary	Heterosexu	LGBA	Don't want	Still waiting	2016 or lat	before 201	MENA	SA	SSA	Other	Total
	n(%)	n(%)	n(%)	n(%)	n(%)	n(%)	n(%)	n(%)	n(%)	n(%)	n(%)	n(%)	n(%)	n(%)
**Limited in whom to have an intimated relation with**														
no	272(50,8)	475(48,3)	8(23,5)	554(50,7)	67(35,6)	92(43,2)	59(34,9)	406(48,1)	266(53,6)	457(48,6)	112(39,7)	161(48,2)	27(62,8)	757(47,3)
I limit myself	125(23,3)	206(20,9)	10(29,4)	253(23,2)	48(25,5)	38(17,8)	35(20,7)	192(22,7)	106(21,4)	202(21,5)	51(18,1)	103(30,8)	7(16,3)	363(22,7)
yes family	58(10,8)	89(9)	4(11,8)	104(9,5)	31(16,5)	9(4,2)	17(10,1)	80(9,5)	53(10,7)	94(10)	31(11)	27(8,1)	4(9,3)	156(9,8)
yes country fello	32(6)	67(6,8)	5(14,7)	65(6)	29(15,4)	8(3,8)	25(14,8)	54(6,4)	24(4,8)	64(6,8)	34(12,1)	15(4,5)	0(0)	113(7,1)
yes my religious	67(12,5)	134(13,6)	2(5,9)	161(14,7)	19(10,1)	19(8,9)	22(13)	113(13,4)	70(14,1)	156(16,6)	29(10,3)	30(9)	0(0)	215(13,5)
yes my sexual id	4(0,8)	19(1,9)	2(5,9)	13(1,2)	8(4,3)	4(1,9)	11(6,5)	11(1,3)	5(1)	11(1,2)	12(4,3)	4(1,2)	1(2,3)	28(1,8)
yes my sexual or	3(0,6)	11(1,1)	2(5,9)	3(0,3)	8(4,3)	4(1,9)	7(4,1)	4(0,5)	4(0,8)	8(0,9)	6(2,1)	2(0,6)	0(0)	16(1)
yes my disability	5(0,9)	8(0,8)	0(0)	10(0,9)	2(1,1)	0(0)	1(0,6)	11(1,3)	2(0,4)	9(1)	1(0,4)	2(0,6)	0(0)	12(0,8)
nothing suits me	43(8)	60(6,1)	5(14,7)	67(6,1)	15(8)	25(11,7)	15(8,9)	60(7,1)	36(7,3)	86(9,2)	18(6,4)	13(3,9)	0(0)	117(7,3)
don't know	37(6,9)	103(10,5)	7(20,6)	76(7)	14(7,5)	48(22,5)	34(20,1)	73(8,6)	33(6,7)	71(7,6)	57(20,2)	24(7,2)	5(11,6)	157(9,8)

Table6: The right to satisfying and safe sexual life free from stigma and discrimination

	Women n(%)	Men n(%)	non-binary n(%)	Heterosexu n(%)	LGBA n(%)	Don't want n(%)	Still waiting n(%)	2016 or lat n(%)	before 201 n(%)	MENA n(%)	SA n(%)	SSA n(%)	Other n(%)	Total n(%)
**Satisfaction in sexual life**														
Satisfied	353(65,3)	663(67,5)	19(55,9)	763(69,4)	109(56,5)	118(57)	107(63,3)	586(69,3)	313(62,9)	627(66)	180(64,5)	220(66,5)	33(75)	1060(66,1)
dissatisfied	29(5,4)	140(14,3)	10(29,4)	124(11,3)	41(21,2)	14(6,8)	38(22,5)	82(9,7)	58(11,7)	101(10,6)	63(22,6)	25(7,6)	1(2,3)	190(11,9)
not applicable	159(29,4)	179(18,2)	5(14,7)	213(19,4)	43(22,3)	75(36,2)	24(14,2)	178(21)	127(25,5)	222(23,4)	36(12,9)	86(26)	10(22,7)	354(22,1)
**Last sex in a safe place**														
no	5(2,6)	45(13,6)	2(20)	43(10,1)	8(14,8)	3(6,5)	8(17)	25(8,9)	17(9)	29(8,2)	9(14,8)	17(15,6)	0(0)	55(10,1)
yes	167(85,6)	257(77,6)	6(60)	352(82,6)	40(74,1)	27(58,7)	32(68,1)	224(80)	157(82,6)	286(81)	48(78,7)	82(75,2)	21(95,5)	437(80,2)
can't/ don't war	23(11,8)	29(8,8)	2(20)	31(7,3)	6(11,1)	16(34,8)	7(14,9)	31(11,1)	16(8,4)	38(10,8)	4(6,6)	10(9,2)	1(4,6)	53(9,7)
**Last sex could suggest condom/contraceptives**														
no	42(20,6)	71(19,8)	1(9,1)	93(20,8)	9(14,3)	6(11,5)	14(26,4)	58(19,3)	36(17,7)	72(18,7)	18(27,3)	23(20,4)	2(9,1)	115(19,6)
yes	122(59,8)	219(61)	7(63,6)	280(62,5)	39(61,9)	26(50)	25(47,2)	175(58,1)	134(65,7)	239(61,9)	34(51,5)	64(56,6)	16(72,7)	353(60,1)
can't/ don't war	40(19,6)	69(19,2)	3(27,3)	75(16,7)	15(23,8)	20(38,5)	14(26,4)	68(22,6)	34(16,7)	75(19,4)	14(21,2)	26(23)	4(18,2)	119(20,3)
**Used the following to avoid pregnancy**														
hormonal methc	45(22,8)	53(14,3)	1(7,1)	82(18,6)	11(13,3)	4(8,2)	10(16,7)	47(15,5)	40(19,9)	58(16,3)	8(9,4)	26(18,4)	9(50)	101(16,8)
condom	50(25,4)	193(52)	6(42,9)	193(43,9)	38(45,8)	15(30,6)	28(46,7)	129(42,4)	81(40,3)	141(39,5)	46(54,1)	58(41,1)	10(55,6)	255(42,4)
intrauterine dev	39(19,8)	20(5,4)	2(14,3)	45(10,2)	7(8,4)	7(14,3)	0(0)	40(13,2)	21(10,5)	54(15,1)	4(4,7)	4(2,8)	1(5,6)	63(10,5)
natural family pl	47(23,9)	49(13,2)	1(7,1)	83(18,9)	8(9,6)	3(6,1)	1(1,7)	51(16,8)	40(19,9)	83(23,3)	3(3,5)	10(7,1)	2(11,1)	98(16,3)
emergency cont	7(3,5)	17(4,5)	0(0)	22(4,9)	1(1,2)	1(2)	3(4,9)	11(3,6)	7(3,4)	8(2,2)	3(3,5)	12(8,2)	0(0)	23(3,8)
no but was neec	7(3,6)	19(5,1)	0(0)	19(4,3)	3(3,6)	2(4,1)	6(10)	8(2,6)	8(4)	11(3,1)	5(5,9)	10(7,1)	0(0)	26(4,3)
no I was plannin	43(18,1)	40(9,8)	1(6,7)	67(13,4)	4(4,7)	6(10,9)	4(6,3)	45(13)	33(14,3)	50(12,4)	6(6,6)	21(13)	3(14,3)	80(11,8)
no I don't know	4(2)	4(1,1)	0(0)	7(1,6)	1(1,2)	0(0)	0(0)	5(1,6)	3(1,5)	7(2)	0(0)	1(0,7)	0(0)	8(1,3)
no we had sex w	14(7,1)	36(9,7)	4(28,6)	41(9,3)	9(10,8)	5(10,2)	6(10)	33(10,9)	18(9)	38(10,6)	9(10,6)	10(7,1)	0(0)	57(9,5)
unsure/don't kn	13(6,6)	49(13,2)	1(7,1)	31(7,1)	16(19,3)	13(26,5)	13(21,7)	26(8,6)	18(9)	20(5,6)	17(20)	25(17,7)	2(11,1)	64(10,7)
other	6(3,1)	11(3)	2(14,3)	11(2,5)	7(8,4)	2(4,1)	3(5)	8(2,6)	9(4,5)	10(2,8)	3(3,5)	8(5,7)	0(0)	21(3,5)
**Used protection against STDs**														
condom	38(16,5)	193(47,5)	8(44,4)	175(35,1)	36(41,9)	23(40,4)	32(49,2)	124(36,3)	69(29,7)	120(30,4)	49(53,9)	59(36,2)	11(52,4)	239(35,7)
no not importan	89(38,5)	62(15,3)	4(22,2)	127(25,5)	13(15,1)	11(19,3)	6(9,2)	86(25,2)	59(25,4)	116(29,4)	8(8,8)	36(22,1)	2(9,5)	162(24,2)
no but was neec	10(4,3)	42(10,3)	2(11,1)	40(8)	10(11,6)	5(8,8)	8(12,3)	26(7,6)	16(6,9)	27(6,8)	6(6,6)	19(11,7)	0(0)	52(7,8)
no I was plannin	37(16)	27(6,7)	1(5,6)	50(10)	4(4,7)	7(12,3)	3(4,6)	34(9,9)	28(12,1)	43(10,9)	3(3,3)	14(8,6)	6(28,6)	66(9,9)
no I don't know	5(2,2)	10(2,5)	0(0)	13(2,6)	2(2,3)	0(0)	1(1,5)	8(2,3)	7(3)	10(2,5)	2(2,2)	4(2,5)	0(0)	16(2,4)
no we had sex w	40(17,3)	36(8,9)	1(5,6)	65(13)	6(7)	5(8,8)	3(4,6)	48(14)	31(13,4)	66(16,7)	2(2,2)	14(8,6)	0(0)	82(12,2)
unsure/don't kn	17(7,4)	48(11,8)	1(5,6)	44(8,8)	15(17,4)	6(10,5)	14(21,5)	26(7,6)	25(10,8)	23(5,8)	23(25,3)	21(12,9)	1(4,8)	68(10,2)
other	7(3)	7(1,7)	2(11,1)	8(1,6)	5(5,8)	3(5,3)	3(4,6)	7(2,1)	5(2,2)	8(2)	3(3,3)	4(2,5)	1(4,8)	16(2,4)
**Discrimination in the last 12 months**														
No	401(73.2)	603(59.8)	24(70.6)	684(62.7)	110(56.4)	158(74.2)	71(40.1)	594(68.5)	333(66.3)	591(62.0)	146(50.2)	264(76.3)	37(84.1)	1038(63.5)
Yes, once	57(10.4)	156(15.5)	1(2.9)	155(14.2)	32(16.4)	20(9.4)	37(20.9)	114(13.2)	60(12.0)	135(14.2)	53(18.2)	41(11.9)	4(9.1)	233(14.3)
yes, several time	90(16.4)	250(24.8)	9(26.5)	252(23.1)	53(27.2)	35(16.4)	69(39.0)	159(18.3)	109(21.7)	228(23.9)	92(31.6)	41(11.9)	3(6.8)	364(22.3)
**Discrimination based on**														
sexual orientatic	2(1.3)	19(4.6)	5(50.0)	7(1.7)	14(16.3)	5(8.6)	9(8.3)	11(4.0)	3(1.8)	11(3.0)	11(7.5)	6(7.9)	0(0.00)	28(4.7)
sexual identity	6(4.0)	14(3.1)	4(40.0)	13(3.2)	5(5.8)	4(6.9)	8(7.3)	10(3.6)	7(4.1)	14(3.8)	8(5.5)	2(2.6)	1(12.5)	25(4.2)
